# Exploring the applicability of virtual reality- enhanced education on extrovert and introvert EFL learners’ paragraph writing

**DOI:** 10.1186/s41239-022-00334-w

**Published:** 2022-06-09

**Authors:** Farzaneh Khodabandeh

**Affiliations:** Department of Linguistics and Foreign Languages, Faculty of Humanities, Payame Noor University, Tehran, Iran

**Keywords:** Augmented and virtual reality, Distance education and online learning, Extrovert and introvert learners, Social media

## Abstract

During the past decade, Virtual reality (VR)-enhanced education has been adopted as a pedagogically new learning approach to smooth the learning progress. With the rise of VR-enhanced activities, investigating the effect of personality types of EFL learners on their writing performance to use VR-based instruction for learning may be a crucial factor influencing their achievement. This study was an attempt to research the impact of VR- enhanced classes on paragraph writing of extrovert and introvert English foreign language (EFL) Learners. To achieve the purpose of the study, first, the Preliminary English Test (PET) was administered for ensuring the homogeneity of the sample. Once the homogeneity was established, a total of 52 EFL intermediate students were selected and placed into two comparative and two control groups, with 13 participants in each group. Four groups took part in the study, with two groups undergoing treatment with the non-VR teaching approach of paragraph writing and two groups undergoing treatment of VR-enhanced education. Among these four groups, two consisted of introverts and two extroverts that were identified through Eysenck Personality Inventory. The two comparative groups received 12 sessions of VR-enhanced education in online classes (instead of drawing pictures and sharing them within their group, they watched the process activities in the VR environment.), but the control group received learning writing paragraphs through using instructor’s instructional materials. After 6-weeks of treatment sessions, all participants of the study took the post-test. According to the results, the VR-enhanced education was more effective than the non-VR teaching approach in developing paragraph writing of both introverts and extroverts. Moreover, the results of the research showed no significant differences between the performance of the introverts and extroverts, meaning that both had the same performance. The results of this study may pave the way for teachers to use VR-based technology in online and traditional classes without worrying about how learners with different personality traits respond.

## Introduction

The application of educational technology tools has become a crucial part of the learning process in and out of classes. In the words of Su and Zou (2020), the use of appropriate technological supplies can be a handful for learners, teachers, and researchers as their use paves the way for chances of collaboration, creation of virtual environments, and development of learner autonomy (Bolgün & McCaw, 2019).

The constant development and improvement of technology have revolutionized the education system and have made it possible for students to use different tools. One of these technologies which is incorporated recently in English foreign language (EFL) teaching and learning is virtual reality (VR) which has attracted the attention of many scholars (Chen, 2016). VR uses a three-dimensional environment (Dickey, 2003) which blends reality with virtual components (Peterson, 2006). VR-enhanced activities revivify the instructional-learning contexts and improve students’ language skills and subskills (Morélot et al., 2021). Application of 3D learning materials in education can be helpful for teaching the curricular subjects where it is important to visualize them (Makransky & Petersen, 2019). VR can increase students’ social interaction (Tang et al., 2016), enable them to develop their creativity (Yang et al., 2018), and provide an opportunity for them to be engaged in authentic learning activities (Chang et al., 2010). Fostering learners’ autonomy (Yeh & Lan, 2018) and reducing their anxiety in another language (Lin & Wang, 2021) can be among the benefits of teaching and learning in the virtual world. In VR instructional-learning environment, learners can be subjected to authentic target language texts in a simulated language condition (Schwienhorst, 2002).

Writing involves complex process skills and students can benefit from using appropriate technology to help them master these skills (Zhang & Zou, 2021). As learners with different learning personality types approach learning differently (Balakrishnan & Gan, 2016), with the latest development of educational technologies, investigating the impact of learners’ personality types on their intentions to use technology for learning has become increasingly important. Accordingly, the complexity of writing and teaching this skill through VR-enhanced activities raised the question whether learners with different personalities (introvert and extrovert) have the same writing achievement or not.

Writing as a “uniquely personal form of individual expression” (Uso’Juan & Martínez-Flor, 2006, p. 473) may be affected by educational, sociological, and personality factors. Students have different personality traits including extroversion, and introversion which underpin the view that their personality variables influence how they effectively learn a second/foreign language (SL/FL) (Liyanage & Bartlett, 2013). Introvert SL/FL learners are those who are unwilling to interact with their peers and teacher in classroom activities. Meanwhile, extroverts are those who are interested in engaging with the tasks involved in language learning (Ehrman et al., 2003). Extroverts experience the world more through social interaction (Xu et al., 2020) while introverts are introspective and more interested in their own thoughts (Zhang, 2008). Extroverted FL learners seem to be good at speaking skill, whereas, FL introverts have less difficulty in writing than extroverts and can express themselves through writing better than extroverts (Boroujeni et al., 2015).

A growing interest in how personality traits have a major effect on SL/FL acquisition process can be observed. Many scholars believe that extroverts are successful EFL learners as they are sociable and engage in inside or outside classroom activities (Caspi et al., 2006). Wang et al. (2009), however, rightly point out that introverts are better learners as they use metacognitive and cognitive strategies. Some students and teachers still are not aware of the important role that their personality traits and technology, particularly VR, play in fostering their general knowledge and specifically their English knowledge. Further, there is a lack of research on the interaction between use of VR and students' personality traits, and how this effects their performance (if at all). Furthermore, the previous studies conducted on writing and personality have not taken into account VR-enhanced education. Most of these studies have compared just two different groups with different personality traits, but not the way they respond to different treatment methods. However, research is scanty on how VR- enhanced classes can impact paragraph writing of extrovert and introvert EFL Learners. Thus, to fill such a gap in the literature and to find out a practical writing method to overcome the common writing problems of learners with various individual differences the current study was an attempt to answer the following questions:

1. Is there any significant difference between the effects of VR- enhanced education and the non-VR teaching approach on EFL learners' paragraph writing?
2. Are there any differences between the effects of VR- enhanced education and non-VR teaching approach on extrovert learners’ paragraph writing?
3. Are there any differences between the effects of VR- enhanced education and non-VR teaching approach on introvert learners’ paragraph writing?

## Review of literature

### VR

The constant development and improvement of technology have revolutionized the education system and made it possible for students to use different tools. VR is one of these tools which plays an important role in this revolutionary process. VR is defined as a realistic 3D virtual world created with the help of a computer that allows players or learners to explore and interact within this environment (Shih, 2014). With the help of VR in the education process, students can engage, explore and build knowledge about places and situations that cannot be explored physically in person (Shih, 2015). The multi-sensory nature of VR makes it possible that information is understood from more than one sense (Radianti et al., 2020).

Immersive VR technologies are used increasingly in education (Hamilton et al., 2021; Marks & Thomas, 2021) which strengthens learners’ constructive process, and reduces their ambiguity and confusion (Broekens et al., 2012). VR- based education is significant in increasing learners’ motivation, engagement, visual and physical immersion (Chen & Hsu, 2020). Huang et al.’s study (2010) revealed that students have more positive attitudes towards VR platforms as they increase their problem-solving abilities. By the use of VR-enhanced activities, teachers can provide contexts that allow learners to experience situations instead of imagining them (Taguchi, 2021) and also benefit the origins of content materials (Roed, 2003).

Many scholars have demonstrated the advantage of VR-enhanced education over traditional methods of teaching (e.g., Crosier et al., 2000; Degli Innocenti et al., 2019; Song & Lee, 2002). According to them, in the VR world, students have the opportunity to experience subject matters and objects that would be difficult if not impossible to illustrate or describe with conventional methods. Moreover, most students remember what they have learned in a VR environment better than what they have learned in other educational centers (Nadan et al., 2011). Different scholars have tried to investigate the role that VR can play in EFL language learning and teaching. For instance, Allcoat and Mühlenen (2018) investigated the effect of educating via VR on learners’ performance, emotion, and engagement and state that learners in the VR class show better performance than those in the traditional class. Besides that, teaching through VR tools increases students’ positive emotions and decreases their negative emotions. VR instructional programs establish holistic problem-based contexts through simulation that can support active and situated learning (Van Ginkel et al., 2020). Moreover, learners’ engagements were reported higher in the VR-enhanced classes than those in traditional ones (Annetta et al., 2009). Likewise, Tseng et al. (2020) and Tai et al. (2020) compared EFL learners’ vocabulary acquisition versus learning via implementing a 3D vocabulary learning tool and indicated that the VR group surpassed the control group as 3D environments develop learners’ autonomy and facilitate their acquisition in a learner-centered context. In another study on learning second language (L2), Xie et al. (2019) unraveled that VR tools develop learners’ oral proficiency as they facilitate learners’ preparation. VR-based education enriches EFL students' confidence, enthusiasm, and willingness to communicate (Ebadi & Ebadijalal, 2020). Lifelong learning and learners’ scaffolding, interaction, and collaboration are the trends of VR-based education (Wang et al., 2020). More to the point, VR-based education creates an immersive environment in which EFL students can interact with a virtual agent in contextualized scenarios and receive feedback on their grammatical mistakes (Morton & Jack, 2005). In a study done by Engwall and Bälter (2007), students received articulation feedback on their pronunciations by a virtual tutor and their attitude toward it was positive as their cognitive awareness of their pronunciation was increased. According to Lamb et al. (2019), exposing learners to both VR tools and textbooks improves their writing ability more than letting them have access just to the VR environment. Learners’ creativity, inner motivation, and writing self‐efficacy are increased more through teaching them with video‐based virtual reality than the group who are taught with the traditional learning method (Huang et al., 2020; Yang et al., 2021). Virtual-assisted writing tools also enhance EFL learners’ expository writing as they develop their spatial ability (Chen et al., 2020). Modern approaches to language learning can be more beneficial than traditional approaches, since traditional approaches often result in passive and disengaged students (Wu et al., 2020).

The literature has also shown that VR-based learning is supported by a social constructivism viewpoint (Tilhou et al., 2020). According to the constructivist perspective to learning, students construct knowledge and their meaning by learning from their experiences (Piaget, 1967). This theory states that individuals make a mental model of the real world based on their experiences with that world. As learners experience new things through VR, they can update their mental models, and construct their interpretation of reality (Vogt et al., 2021). In constructive learning, learners are involved in classroom activities through which they develop the required skills and acquire concepts (Petchtone, 2014). They learn new information by connecting old knowledge to new information and in this way, they add to their prior knowledge (Yakimovicz & Murphy, 1995). Besides that, the constructivists believe that social interaction facilitates learning because students have the opportunity to compare and share their ideas with others through interaction, and as a result, they can learn from others (Wang, 2014). As extrovert learners are mostly social and introverts as less social people, this study creates awareness of social constructivism viewpoint and also lays a foundation for the ways in which extrovert and introvert learners use VR-based technology. Constructivists state that meaningful learning also develops through authentic tasks which will be encountered in real life or being immersed in a VR that does not exist (Alfadil, 2020).

The application of VR-enhanced activities has been incorporated into the language instructional-learning contexts (private institutes), but there has not been a parallel uptake of VR activities in formal language learning settings (state universities and public schools), and it remains unknown in schools and universities. Although different studies accepted affordance, a full-scale search that discussed how they can be applied to EFL learning seemed to be missing in the literature (Wang et al., 2020). Hence, this study aimed to measure the possible impact of VR-enhanced education on EFL learners’ paragraph writing.

### Related studies of personality traits and academic achievements

A few studies have examined the relationship between learners’ personality traits and FL/SL achievement. For instance, De Feyter et al. (2012) investigated the impact of personality traits on learners’ academic performance and concluded that extroversion has a negative relationship with academic performance. By contrast, Cao and Meng (2020), Liyanage and Bartlett (2013) show that extroversion is a strong positive predictor of students’ academic achievement in second language learning. Regarding speaking, much works has been done on the relationship between personality types on EFL speaking. In a study, in 2000, Dewaele and Furnham found in their research that extroverted students as they do not lack self-confidence and can be active to speak in their classes achieve greater fluency in communication tasks compared to introverts. According to Vaezi et al. (2014), extroversion is an essential factor in the development of the speaking ability of EFL learners which requires face-to-face interaction. Extraversion accounts for the largest significant effect on EFL learners’ speaking achievement (Kao & Craigie, 2014). Extroverted EFL learners also excel in group work and communicating (Kappe & van der Flier, 2010), and use foreign language more often than introverted learners (Kao & Craigie, 2014) partially because anxiety towards foreign language learning is more common among introverted learners than extroverts (Dewaele, 2002). Pulford and Sohal (2006) revealed that introverted female learners have lower confidence in speaking skills than extroverts. They engage better in reading and grammar tasks than extroverts (Brown, 2000). By contrast, more recent studies conducted by Arniatika (2020), Nurmayasari and Rahmawati (2016) which examined the relationship between extrovert-introvert learners’ personality traits and their speaking skills, show that here is no difference between extroverted and introverted learners toward their speaking ability. Taking into account the relationship between students’ personality and listening skills, according to Mall-Amiri and Nakhaie (2013), introverts use better listening strategies than extrovert counterparts and their results confirm that personality is an important factor that affects English proficiency among EFL learners. Likewise, Travolta (2018) investigated the achievement of EFL introvert and extrovert students in listening tasks and confirmed that introvert students have better performance in English listening scores than extrovert ones. Training based on personality is also related to the reading ability of EFL learners. For example, Nurianfar et al. (2014) indicated that extrovert EFL learners perform significantly better in reading tasks than introverts as they use more learning activities and study with their friends. Some other researchers have investigated the effect of personality types on EFL writing, for instance, Chamorro-Premuzic and Furnham (2003) stated that extraversion is not related to students’ achievements in essay writing. Similarly, Zainuddin, (2016) confirmed that there is a difference between introverts and extroverts in narrative writing and the former considerably outscore their extrovert peers on the post-test writing. Likewise, Baradaran and Alavi (2015) investigated the impact of introversion and extroversion dimensions on learners' cooperative writing and concluded that introverts outperform extroverts in their writing assignments.

As can be seen, some studies have demonstrated a clear correlation between extroversion and success in acquiring FL/SL (e.g., Cao & Meng, 2020; Dewaele & Furnham, 2000; Kappe & van der Flier, 2010; Liyanage & Bartlett, 2013; Vaezi et al., 2014); still other studies have reached the conclusion that introverts are better learners (e.g., Brown, 2000; Mall-Amiri & Nakhaie, 2013; Travolta, 2018). However, a number of studies have failed to confirm that there is any connection between learners’ personality type and language learning (e.g., Arniatika, 2020; Nurmayasari & Rahmawati, 2016). The brief review of the literature has shown that there exists a controversy regarding the effects of extroversion and introversion on FL/SL learning.

With the rapid growth of VR-enhanced education, the main concern is how to design a VR-based instruction for learning which takes into account learners’ personality differences. Regarding the previous studies, to date, there has not been an empirical study exploring one important issue, how VR- based enhanced education can improve paragraph writing of EFL learners with different personality traits, introverts, and extroverts. So, the current study could shed some light on this area of research.

## Methodology

### Research design

**Fig. 1:**
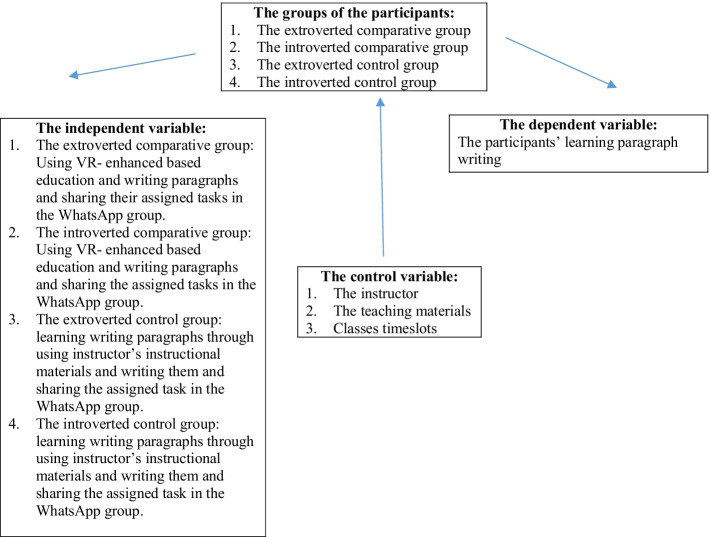
The variables

In this study quasi-experimental research was used. The independent variable which caused the effect on the dependent variable was VR- enhanced based education, and the dependent one that was being measured was the EFL learners’ paragraph writing. This research compared four separate groups; including, one extroverted and one introverted group for the control and the same for the comparative groups. This study was held during the second semester of the academic year of 2021. It lasted for six sessions. Each session was held twice a week (Fig. 1).

### Participants

The participants of the study consisted of 80 male and female learners who were studying English translation at university. They were required to take the two-credit obligatory course of FL Paragraph Writing in the third semester of the 2020–2021 academic year. For homogenization sake, the Preliminary English Test (PET) was administered and 52 learners at the intermediate level of proficiency that ranged in age from 18 to 24 years old were selected. The researcher selected those whose scores were around the mean. It should be mentioned that to identify the participants’ personality types, first, the Eysenck Personality Inventory (EPI) questionnaire was distributed among them and based on the scores of the questionnaire and PET, the selected participants were randomly divided into four groups; two groups (13 introverts and 13 extroverts) undergoing treatment with the non-VR teaching approach of paragraph writing and two groups undergoing treatment of VR-enhanced education (13 introverts and 13 extroverts). They were informed in advance that their identities would be kept anonymous during all phases of the study. They were also informed that the issue of confidentiality would be observed.

### Instruments

Three instruments were used in this study as follows:

#### Preliminary English Test (PET)

To check the homogeneity of the participants, the English language proficiency test called Preliminary English Test (PET) which is one of the standardized tests (Safdari & Fathi, 2020; University of Cambridge ESOL Examinations, 2009) among the series by Cambridge ESOL was used. In the first part of the test, a total of nineteen listening comprehension items are presented. In the second part, students answer twenty-five grammar items. The third part consists of twenty-five items on vocabulary, and in the fourth part, students are supposed to answer twenty-two reading comprehension questions. In the speaking part, which is administered in 10–12 min, participants take the test with a partner (another student). The reliability of the PET was estimated through Cronbach Alpha and it achieved an alpha coefficient level of 0.86 which indicates a high level of reliability.

The speaking and writing rubrics which were used to rate the participants’ writing and speaking sections of PET was provided by Cambridge under the name of the General Mark Scheme. The writing rating was done based on the criteria stated in the writing scale; such as *content, communicative achievement, organization and language*. The speaking rating was done based on *grammar, vocabulary, discourse management, pronunciation and interactive communication*.

For scoring the writing and speaking parts of the PET, besides the instructor herself, there was another rater; a university associate professor holding a Ph.D. in TEFL; who helped the instructor to avoid the subjectivity of scoring. In order to make the dimensions of the rating scale distinct for the other rater, she was trained to use the researcher’s rating scale and provided with detailed description of the criteria stated in the writing scale. The rater was a faculty member of Payame Noor University, and had similar experience of teaching writing courses.

All the writing papers and speaking tests assigned by the two raters were calculated using Pearson Product Moment Correlation (r) formula. The computed Pearson correlation coefficient for coding the writing; such as *content, communicative achievement, organization and language* were 0.89, 0.77, 0.86, and 0.89 respectively, and inter-rater reliabilities for coding the speaking tests done based on *grammar, vocabulary, discourse management, pronunciation and interactive communication* were 0.83, 0.79, 0.79, 0.88, and 0.83, respectively. As can be seen from the results, the deviation ranges from 0.77 to 0.89 which is acceptable in subjective evaluation of writing and speaking skills (Kuiken & Vedder, 2014) and the two raters were fairly consistent in their overall ratings.

#### Writing pre and post-tests

For evaluating the participants' initial paragraph writing skills, pre and post-tests were used. The topics of both tests were selected from the *Advanced writing* book (Hemmari & Khodabandeh, 2019). For the pre-test, the participants spent about 40 min to write at least 200 words about the topic “how to make your favorite food”, and the topic of the post-test was “how to donate blood”. Both tests were rated by two English teachers. The raters scored the participants’ writing ability based on nine criteria according to Hemmati and Khdabndeh’s (2019) model, which consists of *a topic sentence, support sentences, concluding sentence, transitional expressions, cohesion, coherence, formatting a paragraph, completeness, and elements of a process paragraph*.

The scoring procedure of the both pre and post- paragraphs was done by two raters. Pearson correlations was used to identify the inter-rater reliability. Results indicated a noticeable correlation between the two raters who rated the participants’ performance on written paragraphs for pre-test [r (86) = 0.88, which represents a large effect size, p = 0.00] and post-test [r (86) = 0.83, representing a large effect size, p = 0.00].

#### Teaching material

The instructional book used for teaching process paragraph writing was*, Advanced Writing *(Hemmati & Khdabndeh, 2019). The first three chapters of the book which consist of basic concepts and strategies for paragraph writing and the fourth chapter of the book which is about the writing process paragraph were taught in the present research.

#### VR

After getting consent from the dean of the university and consultations with the board of the Isfahan Central Game Center, the virtual reality app was developed to enhance students’ paragraph writing.

Six simple games related to process paragraph writing such as *making banana shakes, organizing a wedding, making a cake, losing weight*, *making a kite,* and *getting a driver’s license*, were developed by the researcher in collaboration with the game developers at Isfahan Central Game Center. The designed games were compatible with the participants’ smartphone platforms with either an iPhone or Android smartphone. The participants were asked to install a VR app, attach their phone to the compatible VR headset and put it on. Each VR game placed the participants inside an experience and immerse themselves and interact with 3D worlds. The participants could tilt their heads and even spin around completely to see more of the virtual world being drawn for them. In the game, the participants could interact with the objects they saw on the VR screen, for example for writing a paragraph about making a cake, within the VR environment they could pick all the ingredients which were placed on the table, stir together the dry ingredients, add eggs and pour the mixture into pans and bake. It should be mentioned that, when they were playing the game, VR superimposed texts into the screen such as verbs, and names of the materials. After playing with the VR app, the participants were asked to write their first draft after obtaining some information in the VR environment.

#### EPI

The EPI is designed to measure the personality traits of learners in terms of being introverted or extroverted. This instrument consists of 57 yes/no items. The scoring is simple, the more “Yes” answers provide the more extroverted the learner is, the same thing for “No” and introversion. The Cronbach Alpha reliability of the personality inventory has been reported as 0.85. Two personality professors confirmed the content and face validity of the EPI.

#### WhatsApp

The messaging service WhatsApp was used in the present study to deliver the course content to the participants of all groups. It is a social-networking application which represents mobile -based communication and allows its users to send free text messages, pictures, audio files and videos to each other. Its practicality and capacity for supporting ubiquitous learning and promoting interaction between EFL learners, their peers and teacher has been stated by many researchers (Andujar & Salaberri-Ramiro, 2021; Tragant et al., 2020). It should be mentioned that WhatsApp is one of those applications which is available in Iran without connecting to a VPN.

The instructor created four separate online groups in WhatsApp named A: the extroverted comparative group and B: the introverted comparative group, C: the extroverted control group, D: the introverted control group and asked the participants of all groups to install WhatsApp on their cell phones, tablets or laptops and join their own groups via the sent invite link.

### Treatment sessions

#### Treatment of the control groups

As previously mentioned, there were two control groups. The participants of one group were extroverts and the participants of another one were introverts. The treatment of both control groups was the same. Because of the COVID-19, all classes were held through a social platform named the WhatsApp application. As such, the instructor first created two separate groups on WhatsApp and added 13 extrovert participants in one group and 13 introvert participants to the other group. The treatment sessions for the control groups were held on Saturdays and Wednesdays of the week from 4:00 to 6:00 p.m. for the extrovert group and 6:30 to 8:30 for the introvert group. During each session, to teach the participants how to write process paragraphs, the instructor taught them the basic parts of a paragraph such as *how to write a topic sentence, support sentences, concluding sentence, transitional expressions, cohesion, coherence, formatting a paragraph, completeness, and elements of a process paragraph.*

**Fig. 2:**
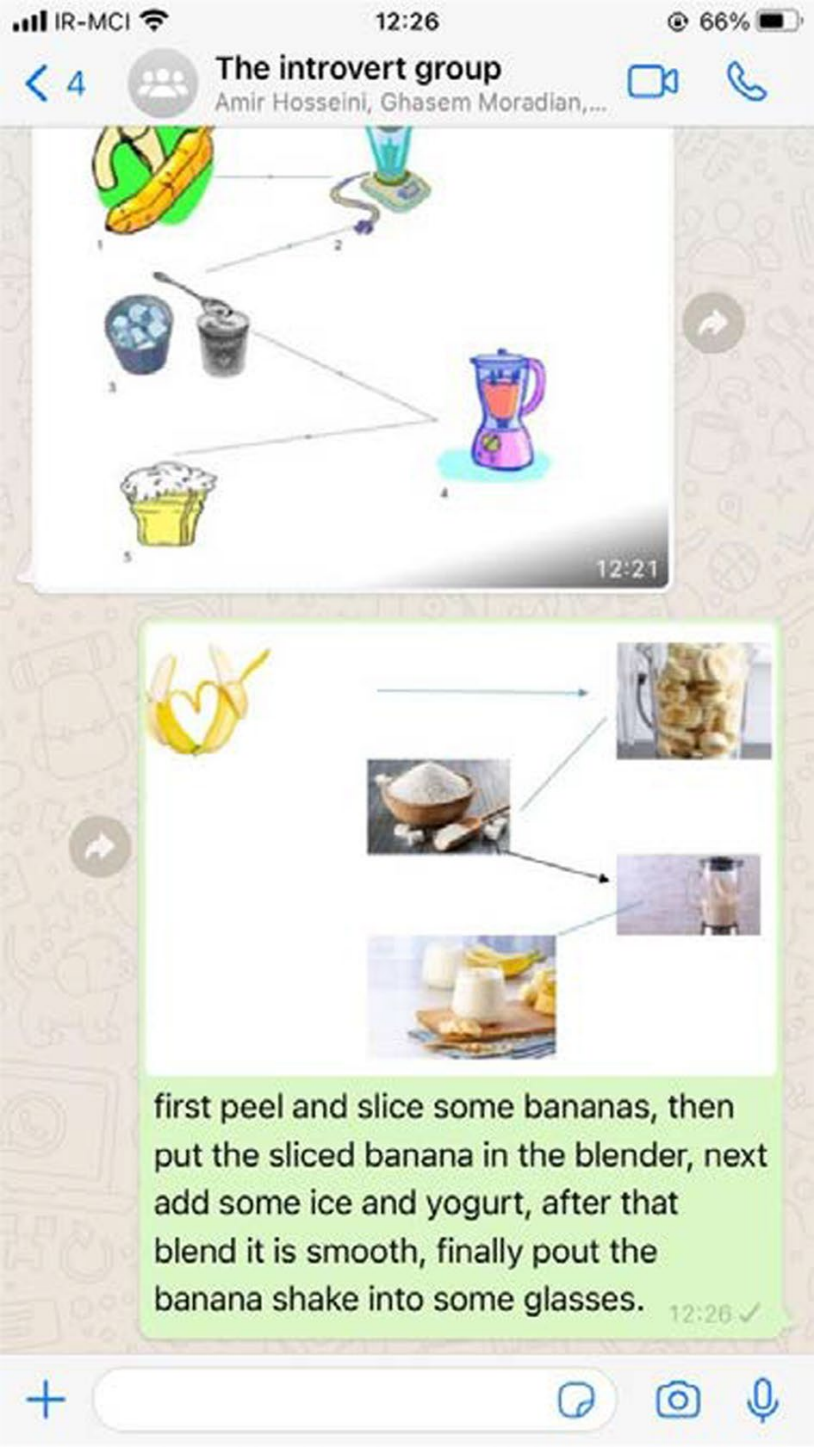
An example of participants’ shared sample within the control group

For the first session, the instructor asked her students in both groups open-ended and thought-provoking questions about the writing process, how the writing process works, why they would need to use the writing process, and how they have used the writing process so far. Before asking the participants to write a paragraph on their own, the instructor taught writing elements and then asked them to define the process paragraph and its different sections and then sent the participants a sample and asked them to name the different parts of the shared paragraph and send their voices to the WhatsApp group. After teaching the basic parts of a paragraph, the instructor gave them a topic such as ‘how to make a banana shake’ and asked them to brainstorm with their classmates within the WhatsApp group and individually write down ideas that might cross their mind and share them within the group and then choose those that are intimately relevant to the topic and important enough to be included in their writing and then they were asked to draw pictures or find some pictures of the process of making a banana shake and send them to their group (Fig. 2).

**Fig. 3:**
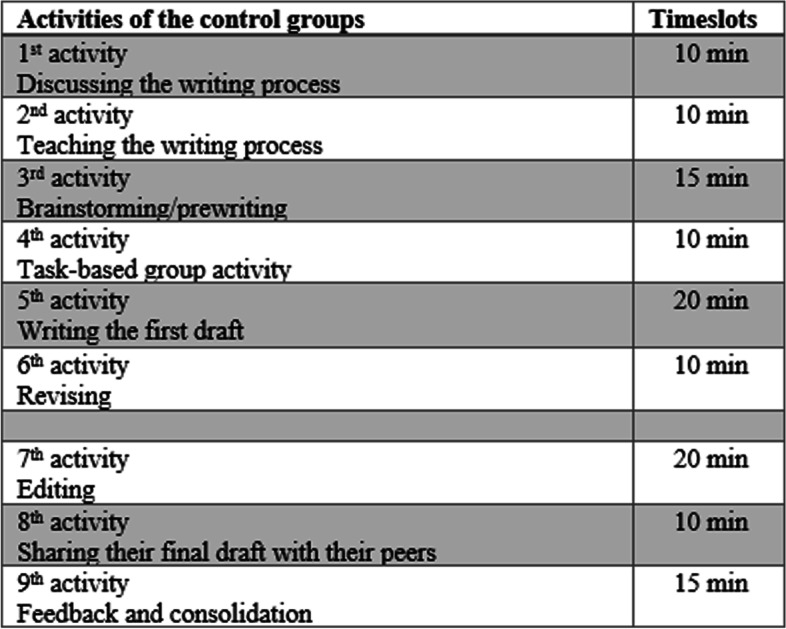
Procedures for the control group

After the brainstorming/prewriting stage, the participants moved on to the drafting stage and were asked to write freely. After 15 min, they were asked to move on to revising their paragraph and include elements like a topic sentence, supporting sentences, and concluding sentence. They were also guided to add or move around other words or sentences and transitional phrases. After revising, they were focused to look at capitalization, punctuation, correct spelling, and grammar. After their final copy was written, the participants were asked to share their paragraphs with their peers within the group. In addition, the instructor let the participants talk about their peers’ paragraphs and share their ideas about them (Fig 3).

#### Treatment of the comparative groups

**Fig. 4:**
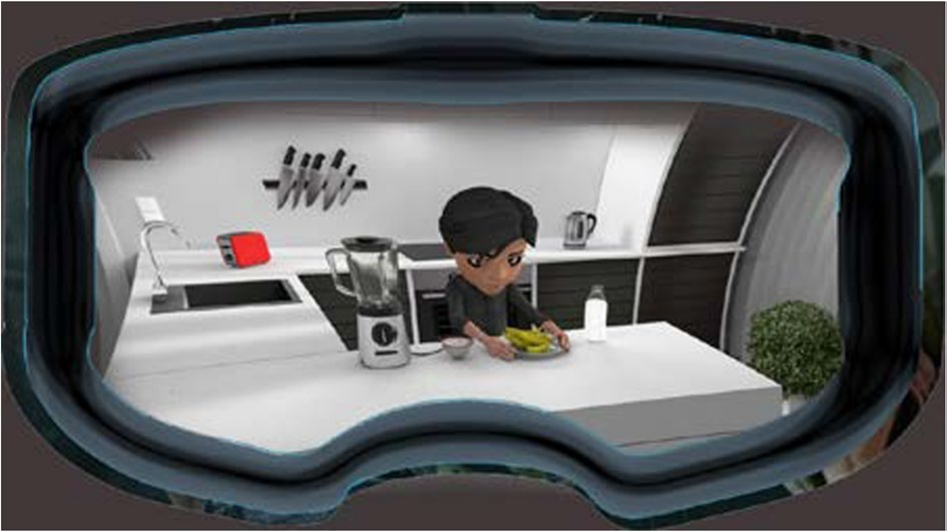
An image of the VR app

Just like the control groups, there were two comparative groups in the current research. The first comparative group was comprised of the introvert participants and the other group was allocated to the extrovert participants. The process of the treatment in these two comparative groups were exactly coincided. The only difference in these groups was about the personality types of the participants. The participants of both groups received 6 sessions working on writing process paragraphs. The instructor first created two separate groups on WhatsApp and added 13 extrovert participants in one group and 13 introvert participants to the other comparative group. The treatment sessions for the comparative groups were held on Sundays and Thursdays of the week from 8:00 to 10:00 a.m. for the extrovert group and 11:00 to 1:00 a.m. for the introvert group. During each session, the instructor taught the participants of both comparative groups the same she taught the control groups. First, she taught the basic parts of a paragraph, then started each class with open-ended and thought-provoking questions about the writing process. After warming up, she taught process paragraph writing elements and then sent the participants a sample and asked them to highlight different parts of the paragraph and send their voices to the group. After teaching the basic parts of a paragraph, the instructor gave them the same topic she gave to the control groups and asked them to brainstorm and write down ideas and then choose those that are relevant to the topic. The only difference between the comparative and the control groups was about using VR applications. During the first session, the instructor asked the comparative participants to launch the VR application on their mobiles; then she taught them how to work with it. After brainstorming, instead of drawing pictures and sharing them within their group, the comparative participants used VR headsets and apps. The VR app helped them to generate ideas about the process of the activity which they were required to write about. For six sessions, the participants watched the process activities in the VR environment. The participants had a remote control that worked with the VR app. With the remote control, they interacted with the objects they saw on the VR screen, for example, they could peel the bananas and pour them within the blender and add some sugar and milk and make a banana shake in a 3D environment. After playing with the VR app, the participants were asked to write their first draft after obtaining some information in the VR environment. The following screen image is an example from the VR environment (Fig. 4).

**Fig. 5:**
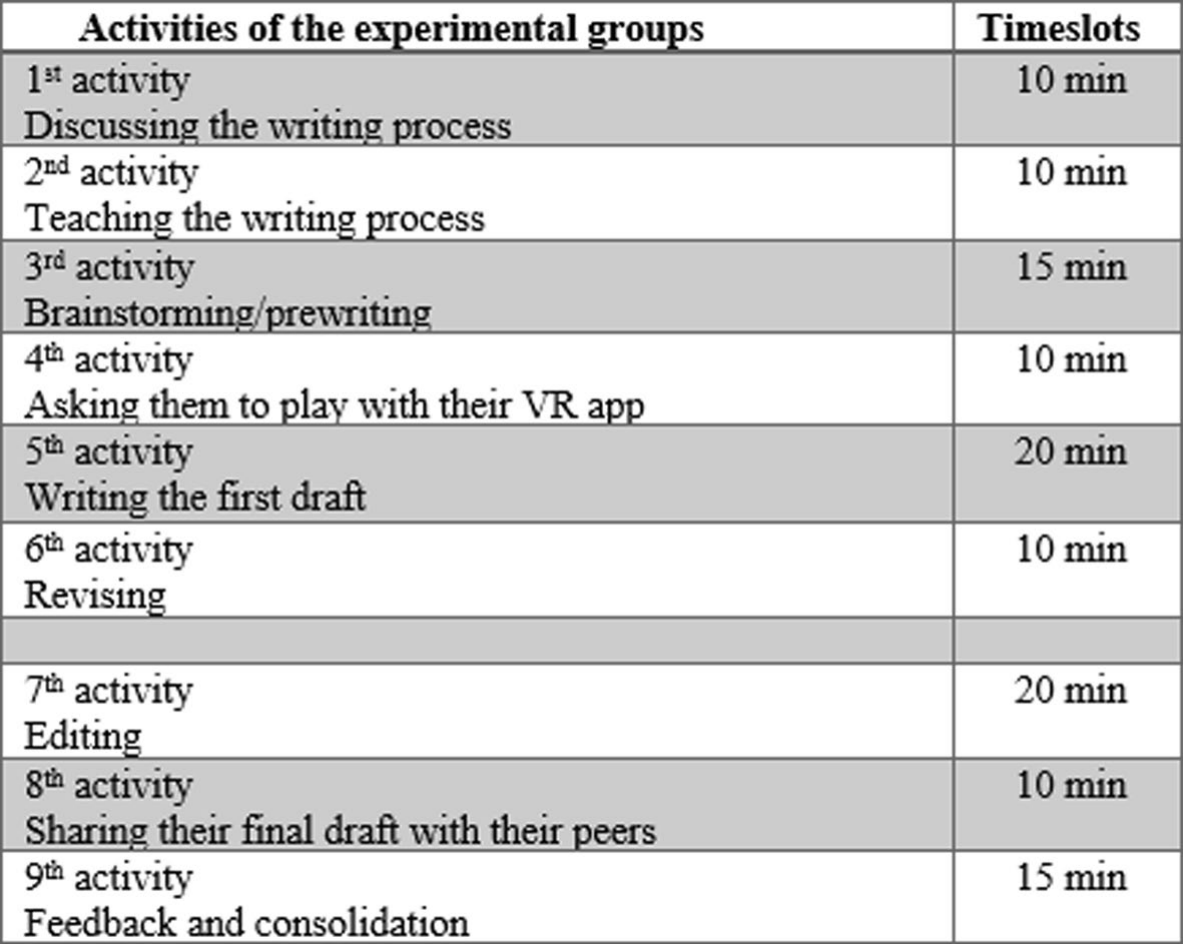
Procedures for the comparative groups

After the prewriting stage, they moved on to the drafting and after 15 min, they were moved on to revising their paragraph, and finally they were asked to write down their final copy and share their paragraphs with their peers within the group (Fig. 5).

## Data analysis

The results of the first research question which is about the efficacy of virtual reality- based education were analyzed through a two- way ANOVA which was run on the pretest and posttest scores of the experimental and control groups to see their progress. The second research question and the third one which are about the effect of virtual reality- based education on the achievement of introvert and extrovert learners were analyzed using ANCOVA. ANCOVA was run on the posttest scores of both groups to measure the effect of the two teaching methods.

To check the normality of the data, the Kolmogorov–Smirnov test was used first.

The Table 1 shows that the data are normal (*sig* > 0.05).

**Table 1:** The results of Kolmogorov–Smirnov test

Groups	Kolmogorov–Smirnov		
	Statistics	DF	Significance
Introvert			
Control	0.161	12	0.20
Comparative	0.151	12	0.20
Extrovert			
Control	0.177	12	0.20
Comparative	0.196	12	0.20

In order to answer the first research question whether there were any significant differences between the effects of VR- enhanced education and the non-VR teaching approach on EFL learners’ paragraph writing; a two- way ANOVA was performed. At first, the results of the pretest were investigated to see whether the groups were similar before the study or not. Descriptive statistics for the scores of the participants on the pretest are summarized in the following table:

As is evident from Table 2, the means of the groups were to some extent equal before conducting the study.

**Table 2:** The results of descriptive statistics for scores of pretests

Personality	Treatment type	Mean	Std. deviation	N
Introvert	Comparative	16.44	0.380	13
	Control	16.35	0.368	13
	Total	16.39	0.374	26
Extrovert	Comparative	16.83	1.253	13
	Control	16.99	0.888	13
	Total	16.91	1.070	26
Total	Comparative	16.63	0.816	26
	Control	16.67	0.628	26
	Total	16.65	0.722	52

According to Table 3, the result of the pretest is not significant at sig > 0.05 (sig = 0.112), meaning that there were no differences between the participants before the study. In other words, they were similar, with no difference whether they were introverts or extroverts.

**Table 3:** Two-way ANOVA for scores on pretest

Source	Type III sum of squares	DF	Mean square	F	Sig
Corrected model	4585.193	3	9170.386	534.65	0.056
Intercept	125,845.324	1	125,845.324	4589.350	0.085
Personality type	26.56	1	28	0.8659	0.223
Treatment type	2895.503	1	1458.6	101.140	0.102
Personality × treatment type	105.502	3	211.004	2.124	0.112
Error	820.07	48	15.084		
Total	128,745.21	52			

To investigate the effect of the treatment and to find the differences between the groups at the end of the study, descriptive statistics and Two- Way ANOVA was run on the post-test scores of all groups.

The Table 4 shows that the means of comparative groups and control groups are different, meaning that the treatment had been effective. In other words, it shows that the VR-based treatment was more effective than the non-VR teaching method in improving the paragraph writing of the participants.

**Table 4:** The results of descriptive statistics for scores of post-tests

Personality	Treatment type	Mean	Std. deviation	N
Introvert	Comparative	18.87	1.208	13
	Control	16..45	0.423	13
	Total	17.66	0.815	26
Extrovert	Comparative	18.93	1.400	13
	Control	16.63	0.398	13
	Total	17.78	0.899	26
Total	Comparative	18.90	1.304	26
	Control	17.11	0.661	26
	Total	17.72	0.857	52

The results of Table 5 show that there is a statistically significant interaction at the sig = 0.012, meaning that the introverts and extroverts responded differently to the treatment methods. However, the table shows that there was no statistically significant difference in paragraph writing regarding personality types (sig = 0.120), meaning that both introverts and extroverts had the same performance on the paragraph writing test. Moreover, there were statistically significant differences between treatment groups (sig = 0.000), meaning that the treatment was effective and had different effects on different personality types, i.e., extroverts and introverts.

**Table 5:** Two-way ANOVA for the scores of the post-tests

Source	Type III sum of squares	DF	Mean square	F	Sig
Corrected model	5525.200	3	900.014	58.200	0.001
Intercept	138,816.600	1	138,816.600	5874.245	0.001
Personality type	28	1	28	1.001	0.120
Treatment type	3245.200	1	1622.6	128.245	0.000
Personality × Treatment type	137.800	1	68.9	2.124	0.012
Error	820.07	34	15.084		
Total	128,745.21	50			
Corrected total	5200.400	49			

To address the second research question whether there were any significant differences between the effects of VR- enhanced education and non-VR teaching approach on extrovert learners’ paragraph writing, an ANCOVA was run on the post-test scores of extrovert learners in both groups.

Table 6 demonstrates that the VR-based approach was significant (sig = 0.06), but the non-VR teaching approach was not significant in improving the participants’ paragraph writing (sig = 0.78). It means that the VR-based environment was more effective in developing the process paragraph writing of the extrovert participants but not the non-VR teaching approach. In other words, it can be said that the VR-based approach was more effective than the non-VR teaching approach in developing the paragraph writing of the extrovert participants.

**Table 6:** ANCOVA on the Post-test scores of extrovert learners in the comparative group and the control group

Variable	Sun of squares	DF	Mean square	F	Sig	Effect%
Extroverts						
Post-test control group	245.05	12	2.003	5.976	0.78	0.09
Post-test comparative group	322.078	12	2.870	7.855	0.06	0.21

To address the third research question whether there were any significant differences between the effects of VR- enhanced education and non-VR teaching approach on introvert learners’ paragraph writing, an ANCOVA was run on the post-test scores of the introvert participants in both the comparative and the control groups.

Table 7 shows that the VR-based approach was significant (sig = 0.043), but the non-VR teaching approach was not significant in improving their paragraph writing (sig = 0.83). It means that the VR-based environment was more effective in developing the process paragraph writing of the introvert participants but not the non-VR teaching approach. In other words, the introvert participants responded better to the VR-based treatment than to the non-VR teaching treatment.

**Table 7:** ANCOVA on the post-test scores of introvert learners in the comparative and the control

Groups						
Variable	Sun of squares	DF	Mean square	F	Sig	Effect%
Introverts						
Post-test control group	2408.02	12	1.087	4.803	0.83	0.09
Post-test comparative group	296.078	12	2.140	6.555	0.043	0.2

## Discussion

The first aim of this study was to determine to what extent VR-based approach could contribute to the development of the paragraph writing of intermediate EFL learners. The mean scores from the learning result of the participants who were treated with VR-approach (18.87 for the introverts and 18.93 for the extroverts) were higher than the mean scores of those treated with non-VR teaching methods (16.45 for the introverts and 16.63 for the extroverts). In other words, the participants who were taught by VR-enhanced education significantly outperformed the participants who were taught by the traditional teacher-centered methods of teaching paragraph writing. Regarding the second aim of the present research, both personality groups had an equal performance, and the interaction between personality type and treatment approach was significant, meaning that both personality types responded similarly in each group. The results showed that the process paragraph writing achievement of the participants in the VR-enhanced education was higher than the process paragraph writing achievement of the participants who were taught with the non-VR teaching approach, although the comparative and control groups received the same amount of time and instruction. Their different performance can be explained by the fact that VR-based environment involved the comparative participants directly in the activity as experiential learning. The VR app used in the present study, gave the participants the opportunities to practice process paragraph writing in a relaxed and pleasurable manner as they visualized real-world through the realistic simulation of the actual world and interacted within that environment. The VR app was a 3D technology that simulated the sequential steps in a procedure leading to its accomplishment. It helped the participants’ visualization of how to do something (for example, how to make a banana shake) which provided them with both interactivity and visual representation. The participants of comparative groups were asked to play the VR app and got enough information required to complete the process and write a process paragraph when they got the guidance for the how-to-do-the process and share it in their group. It enabled them to interact directly with the concepts and made them fully immerse themselves in the context created by the VR app. The results of the present study support the findings of other studies that showed VR-enhanced education increased the participants’ engagement, visual and physical immersion (Chen & Hsu, 2020; Shih, 2014; Taguchi, 2021). Additionally, when the participants were playing the game, it helped them contextualize the learning material which assisted them in comprehending the process within the physical settings that supports Roed (2003) who stated that VR apps help EFL learners achieve knowledge internalization and also benefit the origins of content materials. Therefore, the VR app used in this study enhanced teaching effectiveness for the participants of both comparative groups. Regarding these facts, the results of the study are in line with findings of other studies that showed VR- enhanced education was a helpful activity which helped the comparative participants to visualize the real-world scenes and improve their achievement (e.g., Allcoat & Mühlenen, 2018; Chen et al., 2020; Crosier et al., 2000; Degli Innocenti et al., 2019; Engwall & Bälter, 2007; Huang et al., 2020; Lamb et al., 2019; Nadan et al., 2011; Song & Lee, 2002; Tseng et al., 2020; Tai et al., 2020; Wang et al., 2020; Xie et al., 2019; Yang et al., 2021).

The analysis of the second and the third research questions showed a comparison of the two introvert groups and two extrovert groups and their paragraph writing after the treatment application. There was a difference between the writing result of the extroverted participants in the comparative group and the control group, with the comparative group performing better than the control group, which was evident from their mean scores in the post-test (comparative group = 18.93, control group = 16.63), suggesting the superiority of VR- enhanced education over non-VR teaching approaches to learning.

Like the second research question, the third research question showed a difference between the paragraph writing result of the introverted participants in the comparative and the control groups, with the comparative group performing better than the control group, evidence from their mean scores in the post-test (comparative group = 18.87, control group = 16.45). The extrovert and introvert participants in the comparative group that received the treatment through a VR- enhanced education had better performance and made more progress. In the literature, mixed results are achieved regarding the superiority of extroverts over introverts and vice versa, and therefore, making a distinction between them is difficult. According to Ellis (1994), second language learning and personality types are closely linked. The first is that extrovert learners are more successful language learners because they are better communicators and use more communication strategies. Likewise, introvert learners are also better language learners because they demonstrate higher cognitive academic achievement, which is congruent with the results achieved for the introverts and extroverts of this study, performing the same in both comparative and control methods, meaning that the treatment had different effects on their paragraph writing, and their personality type was not effective.

Although no special studies are conducted on the role of technology in general, and VR- enhanced education in particular in developing process paragraph writing of extroverts and introverts, the results gained about these two personality traits confirm the results of the studies which found no or even negative difference between the performance of the extroverts and the introverts (e.g., Chamorro-Premuzic & Furnham, 2003; De Feyter et al., 2012). However, the results of the current research contradict the results of those studies proving that extroversion may be beneficial for EFL learners, such as Cao and Meng (2020), Dewaele and Furnham (2000), Ehrman (2008), Kao and Craigie (2014), Kappe and van der Flier (2010), Liyanage and Bartlett (2013), Nurianfar et al. (2014), Pulford and Sohal (2006), and Vaezi et al. (2014), and those studies which found introverts to be better learners as compared to the extroverts (e.g., Baradaran & Alavi, 2015; Brown, 2000; Ehrman, 2008; Mall-Amiri & Nakhaie, 2013; Travolta, 2018; Zainuddin, 2016).

## Conclusion

This study explored the impact of VR-enhanced education on students’ personality traits in their paragraph writing. Based on the results, the significant role of the VR-enhanced education in improving the paragraph writing of the introvert and extrovert participants was stated. It can be concluded that there was an interaction between VR-enhanced education and personality in the participants’ paragraph writing. Taking into account the fact that using the VR-enhanced education can be considered as helpful instructional material in teaching paragraph writing to EFL learners, it can be stated that the proper use of VR technology would significantly assist instructors to promote EFL learners’ paragraph writing. The findings for VR- enhanced education and traditional classrooms revealed no significant differences in process paragraph writing based on introversion/ extroversion. Therefore, teachers can employ this specific technology without worrying about what their class is dominantly with, the extrovert or the introvert learners.

Like any other study, the present one has its limitations. Firstly, the sample size was small; as the participants were divided into four groups, each group had just 13 students, and as a consequence, the findings of the study cannot be generalized to other language learners. Secondly, it was conducted on just university learners. The study might be replicated on a larger scale and if possible learners at another level of proficiency.

The study has important pedagogical implications. Firstly, well-planned and designed VR apps can promote EFL learners’ paragraph writing. The results of the current study can help EFL teachers to embed VR technologies in their classrooms to improve EFL learners’ writing. Also, using VR apps in EFL classes can enhance EFL learners’ motivation, enjoyment and also their team-work skills. Teachers need training in using VR apps that is easily available and they should be trained on how to adopt technology in their teaching practices.

## Data Availability

The pre and post tests and the teaching materials are available from the corresponding author on reasonable request.
